# Co‐diagnoses of acute myeloid leukaemia and COVID‐19: presentation and management implications

**DOI:** 10.1002/rcr2.650

**Published:** 2020-08-25

**Authors:** Amy O'Brien, James Campling, Hugh Goodman, Catherina L. Chang

**Affiliations:** ^1^ Department of Respiratory Medicine Waikato Hospital Hamilton New Zealand; ^2^ Department of Haematology Waikato Hospital Hamilton New Zealand

**Keywords:** Acute myeloid leukaemia, COVID‐19, immunosuppression, SARS‐CoV‐2

## Abstract

We report a case of concurrent new diagnoses of confirmed severe acute respiratory syndrome coronavirus 2 (SARS‐CoV‐2) infection and acute myeloid leukaemia (AML). We review the existing literature on coronavirus disease 2019 (COVID‐19) in the immunocompromised patient and the implications for managing our patient's haematological neoplasm. The implications of severe immunocompromise are unclear in the context of infection with SARS‐CoV‐2. Respiratory and viral systemic symptoms remained mild in this patient and this is consistent with the existing literature on COVID‐19 in immunocompromised patients. To our knowledge, this is the first description of a case of SARS‐CoV‐2 infection with AML.

## Graphical abstract

We report a case of concurrent new diagnoses of confirmed severe acute respiratory syndrome coronavirus 2 (SARS‐CoV‐2) infection and acute myeloid leukaemia (AML). The implications of severe immunocompromise are unclear in the context of infection with SARS‐CoV‐2. We describe the clinical course of coronavirus disease 2019 (COVID‐19) in this case, review the existing literature on SARS‐CoV‐2 in the immunocompromised patient, and finally the implications for managing her haematological neoplasm.

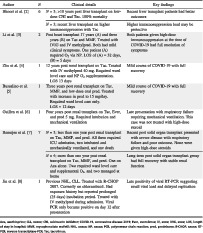

## Introduction

Severe acute respiratory syndrome coronavirus 2 (SARS‐CoV‐2) emerged from the epicentre in Wuhan, China, in December 2019 with rapid human to human transmission. At the time of writing, there have been over eight million confirmed cases with over 450,000 deaths worldwide [1]. As the novel virus moves through the population, it is inevitable that people with concurrent severe illness also become infected. We present a case of confirmed SARS‐CoV‐2 infection in a patient with newly diagnosed acute myeloid leukaemia (AML) and the progress of both illnesses through intensive induction chemotherapy. A review of the existing literature on SARS‐CoV‐2 infection in the immunocompromised patient is also presented.

## Case Report

A previously fit and healthy 54‐year‐old Maori woman presented to a secondary care hospital with a 10‐day history of increasing fatigue and three days of progressive fevers, dyspnoea, headache, myalgias, and dry cough. She denied anosmia. The only significant medical history was pT1c grade 2 breast cancer treated with surgery, adjuvant chemotherapy, and radiotherapy in 2013. There was no family history of haematological malignancy. She was an ex‐smoker with a 15 pack‐year history.

On examination, the patient had pallor with mild tachycardia (115 bpm), low pulse oximetry [90% on Fraction of Inspired Oxygen (FiO_2_) 21%], and tachypnoea (24/min). She required 1–2 L of O_2_ via nasal prongs to maintain O_2_ saturation > 92%. Her complete blood count showed moderate macrocytic anaemia (haemoglobin: 77 g/L) with moderate thrombocytopenia (platelets: 80 × 10^9^/L). There was a marked leucocytosis (white cell count (WCC): 377.1 × 10^9^/L) comprised almost entirely of blasts. Flow cytometry of peripheral blood confirmed myeloid blasts with expression of myeloperoxidase (MPO), CD13, CD33, and with aberrant CD7. Karyotyping showed 46,XX and molecular analysis showed a type D NPM1 mutation, an FLT3 internal tandem duplication (ITD) (high variant allele fraction), and a low‐level FLT3 tyrosine kinase domain (TKD) mutation.

Other key laboratory findings included elevated C‐reactive protein of 130 mg/L, lactate dehydrogenase (LDH) of 2450 U/L, d‐dimer of 16,300 μg/L, and N‐terminal‐pro Brain Natriuretic Peptide (NT‐proBNP) of 263 pmol/L. Serial high‐sensitivity troponins were normal. A portable Anterior‐Posterior (AP) chest radiograph showed no interstitial infiltrates, lobar consolidation, or effusion.

The patient was diagnosed with therapy‐related AML (t‐AML) and admitted to the haematology ward. Although it was felt highly probable that her presenting symptoms were explained by her AML, in line with the broad testing strategy in New Zealand at the time, a nasopharyngeal swab for reverse transcriptase‐polymerase chain reaction (RT‐PCR) for SARS‐CoV‐2 was taken and the patient was isolated until the RT‐PCR results became available. No other source of infection was identified and the patient was commenced on empiric amoxicillin/clavulanic acid. She was then transferred to a tertiary hospital for specialized haematology care and a repeat nasopharyngeal swab for RT‐PCR for SARS‐CoV‐2 was sent on arrival. The swabs were taken 7 h apart. The first swab became positive on day 2 at 35 cycles. This was sent for confirmatory testing to the national reference laboratory and was confirmed positive. The second swab was processed locally at the tertiary centre and this was negative at 38 cycles (local testing protocol). At this point, the tertiary centre laboratory requested and tested an aliquot from the first swab which again became positive at 35 cycles. Following discussion involving a haematologist (H. Goodman), a respiratory physician (C. L. Chang), and a clinical microbiologist, it was deemed that a false‐positive result was unlikely due to a very low false‐positive rate with local RT‐PCR methodology and multiple positive confirmatory tests at different laboratories. The conclusion was that the second sample was a false negative possibly due to suboptimal sampling of the nasopharynx resulting in a nasal or mid turbinate swab. The patient also had a plausible epidemiological risk factor working in a hotel accommodating international guests.

Chemotherapy was commenced despite the positive SARS‐CoV‐2 status due to unacceptable risk of disease progression and mortality without treatment. Cytoreduction was initially achieved with hydroxyurea then cytosine arabinoside. Intravenous dexamethasone was commenced for possible hyperleucocytosis. Rasburicase and intravenous fluid were given to prevent tumour lysis syndrome (TLS). Cytoreduction continued until WCC was <50 × 10^9^/L prior to intensifying to induction therapy with an anthracycline.

The patient continued to express symptoms of only a mild coronavirus disease 2019 (COVID‐19) phenotype. Low‐grade fevers continued until the third day of admission. The patient did not require supplemental oxygen after the initial presentation. Dyspnoea on minimal exertion and dry cough continued until the fifth day of admission where the respiratory rate normalized and no further respiratory symptoms were reported. At this time, cytoreduction had achieved a leucocyte count < 100 × 109/L. Three SARS‐CoV‐2 RT‐PCRs between days 13 and 19 of admission were negative and the patient was successfully de‐isolated to the haematology ward on day 20. During her 26th day of admission, she completed a cycle of induction chemotherapy, had a second course of antibiotics for neutropenic colitis, and was discharged on cell count recovery. Her disease was refractory to daunorubicin, ara‐C (DA) induction chemotherapy but she subsequently achieved morphological remission with fludarabine, ara‐C, granulocyte colony stimulating factor, idarubincin (FLAG‐IDA) salvage and has been referred for consideration of allogeneic stem cell transplant.

## Discussion

This case highlights the potential of coexistence of COVID‐19 and other acute severe diagnoses including acute haematological malignancies, where both the pathophysiology and treatment may render the patient highly immunovulnerable.

There is limited existing literature on the clinical course and outcome of SARS‐CoV‐2 infections in immunosuppressed states, such as patients with active malignancy or due to immunosuppressants. Case reports have shown that the presentation and outcomes can vary from mild to severe disease [2, 3, 4, 5, 6, 7, 8]. As immunosuppressive therapy downregulates the production of interleukins (ILs) and the proliferation, survival, and maturation of T cells, it is possible that although the risk of acquiring the virus is higher, the anti‐inflammatory effects of such therapy reduce the overall severity of COVID‐19 [9]. Furthermore, AML itself leads to impaired T‐cell response [10]. We note evidence supporting the link between overactivation of T cells and severe immune injury in patients with severe COVID‐19 [11]. Systemic inflammatory response has also been shown to play a role in provoking viral‐induced lung injury.

Table 1 summarizes the case reports of COVID‐19 infection in patients who are immunosuppressed as a result of solid organ transplant or Chronic Lymphocytic Leukaemia (CLL). Of the 19 patients described, four died. Of note, three of these four patients were on very low‐dose immunosuppression therapy at the time. The fourth death was unrelated to COVID‐19. In terms of severity of disease, five patients were managed as outpatients; seven were admitted, one of whom did not have an oxygen requirement, and four required intensive care unit (ICU) for ventilation, one of whom was successfully de‐escalated to ward‐based care. Of note, ours is the first reported case of a patient with COVID‐19 actively undergoing cytoreduction chemotherapy rather than broad‐spectrum immunosuppression following transplantation.

**Table 1:** Summary of all case reports of COVID‐19 infection in patients who are immunosuppressed as a result of solid organ transplant or CLL.

Author	*N*	Clinical details	Key findings
Bhoori et al. [2]	6	*N* = 3; >10 years post liver transplant on low‐dose CNI and Tac. 100% mortality	Recent liver transplant patients had better outcomes
		*N* = 3; recent liver transplant on higher immunosuppression with Tac	Higher immunosuppression load may be protective
Li et al. [3]	2	Post heart transplant 17 years (A) and three years (B) on Tac and MMF. Treated with IVIG and IV methylpred. Both had mild clinical symptoms. One patient (A) required O_2_ via NP. LOS of (A) = 32 days, (B) = 5 days	Both patients given high‐dose immunosuppression at the time of COVID‐19 had full resolution of symptoms
Zhu et al. [4]	1	12 years post renal transplant on Tac. Treated with IV methylpred 40 mg. Required ward level care and NP O_2_ supplementation. LOS 13 days	Mild course of COVID‐19 with full recovery
Bussalino et al. [5]	1	Three years post renal transplant on Tac, MMF, and low‐dose oral pred. Treated with increase in pred to 15 mg/day. Required ward level care only. LOS = 12 days	Mild course of COVID‐19 with full recovery
Guillen et al. [6]	1	Four years post renal transplant on Tac, Ever, and pred 5 mg. Required intubation and mechanical ventilation	Late presentation with respiratory failure requiring mechanical ventilation. This case was not treated with high‐dose steroid
Banerjee et al. [7]	7	*N* = 3; less than one year post renal transplant on Tac, MMF, and pred. All three required ICU admission, two intubated and mechanically ventilated, and one death	Recent post solid organ transplant presented with severe disease with respiratory failure and poor outcome. None were given high‐dose steroids
		*N* = 4; more than one year post renal transplant on Tac, MMF, and pred. One on Aza alone. Two required ward level care and supplemental O_2_, and two managed at home	Long‐term post solid organ transplant group had full recovery with stable renal function
Jin et al. [8]	1	Previous NHL, CLL. Treated with R‐CHOP 2007. Currently on chlorambucil. Had exposure history but reported prolonged (25 days) incubation period. Treated with IV methylpred during admission. Viral PCR only became positive on day 12 after presentation	Late positivity of viral RT‐PCR suggesting small viral load and delayed replication

Aza, azathioprine; CLL, Chronic Lymphocytic Leukaemia; CNI, calcinuerin inhibitor; COVID‐19, coronavirus disease 2019; Ever, everolimus; IV, intravenous; IVIG, intravenous immunoglobulin; LOS, length of stay in hospital; MMF, mycophenolate mofetil; NHL, non‐Hodgkin's lymphoma; NP, nasal prongs; PCR, polymerase chain reaction; pred, prednisone; R‐CHOP, rituximab, cyclophosphamide, hydroxydaunomycin, vincristine, prednisone; RT‐PCR, reverse transcriptase‐PCR; Tac, tacrolimus.

In this case, the patient was given dexamethasone for hyperleucocytosis. At the time of treatment, steroids were not recommended for the management of COVID‐19 as they have not demonstrated efficacy in prior SARS or Middle East respiratory syndrome (MERS) epidemics. The early reports of steroid use from China suggest detrimental effects rather than benefits. However, the recent RECOVERY Trial conducted in the UK found that dexamethasone was associated with improved outcomes in patients hospitalized with COVID‐19 [12]. It is possible that early administration of systemic steroids improved the overall clinical course of the coronavirus infection in our patient.

Elevated serum ferritin and IL‐6 level have been associated with increased mortality from COVID‐19 infection suggesting inflammation may contribute to poor outcomes. It has been suggested that a subset of patients with COVID‐19 and high H‐score (used in the diagnosis of haemophagocytic syndromes) may benefit from immunosuppression with biological agents such as tocilizumab [13]. Thus, patients undergoing intensive induction chemotherapy are less likely to suffer from a cytokine storm, which is a proposed pathway leading to COVID‐19‐related mortality. Interestingly, despite severe immunosuppression, our patient only developed mild respiratory symptoms. Furthermore, the pulmonary and neurological symptoms may also be secondary to AML‐associated hyperleucocytosis—fever and medullary pain can also accompany acute leukaemia which may masquerade as the fever and myalgia of a viral respiratory illness. Other elevated prognostic markers of COVID‐19 such as d‐dimer, LDH, and thrombocytopenia can all be attributed to acute leukaemia in this patient. It was unclear if the clinical symptoms in this case were secondary to hyperleucocytosis or COVID‐19 infection.

The coexistence of a highly communicable infectious disease also introduced significant practical complications in the management of this case. Following careful consideration, the patient was placed in isolation in the respiratory ward rather than the haematology ward to reduce exposure to other vulnerable patients. There is also concern that immunocompromised patients have prolonged viral shedding and may remain infective for longer. We were conservative in de‐isolating our patient to the main haematology ward and only did so following complete resolution of respiratory symptoms and two consecutive negative PCR nasopharyngeal swabs were obtained. Furthermore, isolation for the first few weeks with minimized staff exposure and no family or friend visits coupled with the natural grief process of such a grave diagnosis may lead to an increased need for psychological health support.

Routine monitoring and treatment options were also affected by the co‐diagnoses. For example, we were unable to obtain a pre‐treatment echocardiogram prior to commencing anthracycline due to local infection control guidelines, although we note that myocarditis is a well‐recognized sequelae of COVID‐19 infection [14]. Instead, the patient received the iron chelator dexrazoxane to decrease the risk of anthracycline‐induced cardiotoxicity. We also elected to continue moderately aggressive fluid resuscitation despite an elevated NT‐proBNP and the risk of cardiac complications following COVID‐19, as the benefit of preventing TLS was deemed greater than the potential risks of fluid overload.

In conclusion, this case report adds to the discussion of COVID‐19 and whether immunosuppression alters the clinical course and outcome. It highlights the difficulties of treating high‐grade haematological neoplasia in the current pandemic. To our knowledge, this is the first documented case of AML and COVID‐19.

### Disclosure Statement

Appropriate written informed consent was obtained for publication of this case report and accompanying images.
